# Fumed silica-based composite phase change materials with effective electric and magnetic heating abilities for wearable thermotherapy†

**DOI:** 10.1039/d5ra00438a

**Published:** 2025-04-11

**Authors:** Giang Tien Nguyen, Lam Truong Nguyen, Nhung Tran Thi, Nguyen Thanh Nho, Le Thi Duy Hanh, Huynh Nguyen Anh Tuan, Thanh Vy Nguyen

**Affiliations:** a Faculty of Chemical and Food Technology, Ho Chi Minh City University of Technology and Education (HCMUTE) 1 Vo Van Ngan, Thu Duc Ho Chi Minh City 700000 Vietnam ntgiang@hcmute.edu.vn; b Institute of Applied Technology and Sustainable Development, Nguyen Tat Thanh University 300A, Nguyen Tat Thanh, Ward 13, District 4 Ho Chi Minh City 700000 Vietnam

## Abstract

In this study, paraffin wax (PW) was combined with fumed silica (FS) as a porous support and Fe_3_O_4_-incorporated expanded graphite (EG@Fe_3_O_4_) as a thermal conductivity enhancer and multifunctional thermal conversion agent. This combination resulted in the development of PW/FS/EG@Fe_3_O_4_ composite phase change materials (CPCMs) with varying PW content (60–80%). FS provided ample space to stabilize a significant amount of PW (up to 75%) without liquid leakage. The crystallization fractions of the confined PW exceeded 97%, outperforming most reported values for other PCMs confined in SiO_2_-based materials and enabling high phase change enthalpies (*e.g.*, 146.1 J g^−1^ for the composite with 75% PW). The thermal conductivities of the 60–80% PW CPCMs were significantly enhanced to 2.215–1.395 W (m K)^−1^, representing an increase of 9.8–6.2 times compared to pristine PW. Additionally, EG@Fe_3_O_4_ endowed the CPCMs with electrothermal and magnetothermal conversion capabilities due to the high electrical conductivity of EG and the superparamagnetism of Fe_3_O_4_. Experimental testing of the 75% PW composite demonstrated its ability to exceed its melting point under the application of either a DC voltage or an alternating magnetic field. When used as a heat pack, the 75% PW CPCM maintained a consistent heat release within the 50–55 °C range for 12 minutes on a volunteer's back, meeting and surpassing the requirements for high-temperature thermotherapy. Overall, the combination of high thermal conductivity, substantial phase change enthalpy, excellent cycling durability, and multifunctional thermal conversion makes PW/FS/EG@Fe_3_O_4_ CPCMs highly promising for practical thermotherapy applications.

## Abstract 2

A heat pad produced from PW/FS/EG@Fe_3_O_4_ composite shows a high thermal energy storage capacity. It can be effectively charged by an alternating magnetic field or a DC voltage and shows qualified performance in thermotherapy.

## Graphical abstract

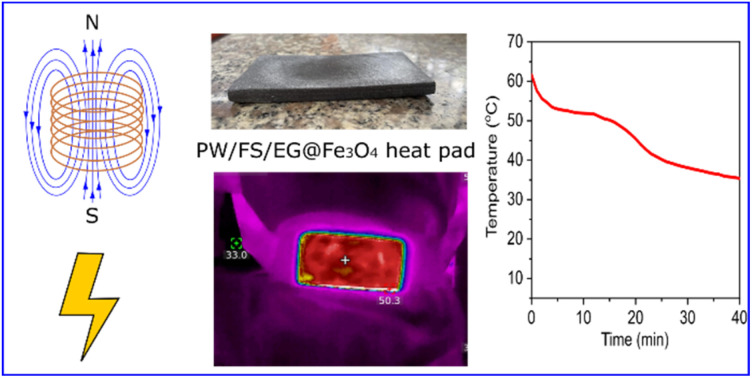

## 1. Introduction

Thermotherapy, a non-invasive therapeutic technique that uses controlled heat to treat various medical conditions, has gained significant attention due to its potential to alleviate pain, enhance blood circulation, and promote tissue healing.^1,2^ The maintenance of therapeutic temperatures is critical to its efficacy and can be categorized into high level (50–55 °C for 4–6 min), moderate level (40–50 °C for 15–60 min), and low level (35–40 °C for 6–72 h).^3,4^ Traditional thermotherapy methods, such as hot water bottles and electric heating pads often face challenges in maintaining stable temperatures over extended periods, limiting their effectiveness and safety in clinical and home-care settings.^5,6^ Advanced methods such as ultrasounds are expensive and require patients not to move during the treatment, which also causes certain limitations.

Phase change materials (PCMs), known for their ability to absorb and release large amounts of latent heat during solid–liquid phase transitions, present an innovative solution to these limitations.^7,8^ PCMs can maintain near-constant temperatures during their phase transition, making them ideal for applications requiring controlled heat delivery. The utilization of PCMs for thermotherapy offers significant advantages, including prolonged heat retention, precise temperature control, adaptability to diverse treatment protocols, and high mobility. Chen *et al.*^9^ prepared a functional facemask incorporated with polyethylene glycol (PEG) PCM, which could provide heat at ∼43.5 °C for ∼33 min for the thermotherapy of allergic rhinitis. Lin *et al.*^6^ demonstrated that a wearable thermotherapy pack based on paraffin PCM maintained a temperature above 40 °C for 30 min when applied to a volunteer. Similarly, Shao *et al.*,^10^ Chen *et al.*,^1^ and Zhang *et al.*^11^ demonstrated effective thermotherapy methods based on various PCMs. However, pure PCMs are subjected to liquid leakage during solid–liquid phase transitions. Therefore, they always need to be stabilized by impregnation into porous structures or encapsulation in core–shell systems.^8,12^

Fumed silica (FS) has been commonly utilized as a porous support for stabilizing various PCMs for its high PCM adsorption capacity (70–80%), low cost, high availability, and non-toxicity. The biggest disadvantage of FS-based composite phase change materials (CPCMs) is the low thermal conductivity and infeasible thermal conversion ability, limiting their thermal charging performance.^13–15^ Indeed, for thermal charging, the FS-based CPCMs need to be placed in elevated-temperature environments such as heated air or heated water, which causes inconveniences or even damages due to the low convection heat transfer of the hot air and the high permeability of hot water. Currently, thermotherapy devices possessing multifunctional thermal conversion abilities (electrothermal and magnetothermal conversion) exhibit highly desired features due to facile usage and effective charging performance. To possess these advanced functions, energy converters (electrothermal and magnetothermal converters) need to be added to CPCMs. Lin *et al.*^6^ took advantage of the high thermal and electrical conductivity of expanded graphite (EG) to equip an accelerated electrothermal conversion for paraffin PCM. Gao *et al.*^16^ employed Fe_3_O_4_-anchored Mxene as a magnetothermal converter for PEG PCM owing to the superparamagnetic nature of Fe_3_O_4_. Shen *et al.*^17^ employed a carbon aerogel@Fe_3_O_4_ as an electrothermal and magnetothermal converter for PEG PCM, achieving multifunctional thermal conversion and storage. Alternatively, graphene,^18^ graphene aerogel,^19^ Co nanoparticles/MOF/carbonized melamine foam,^4^ and polypyrrole-decorated melamine foam (PPy@MF)^1^ have been utilized as electro/magnetothermal converters in various CPCMs. In the above-mentioned reports, however, the single thermal conversion, *i.e.*, electrothermal conversion, of EG, PPy@MF, graphene, graphene aerogel, and the high cost of multifunctional Fe_3_O_4_-anchored Mxene and Co nanoparticles/MOF/carbonized melamine foam thermal converters may greatly limit their practical applications in thermotherapy.

In this work, we demonstrated a facile and low-cost strategy to prepare advanced multifunctional FS-based CPCMs with high thermal conductivity and effective electro/magnetothermal conversion and storage ability for thermotherapy. We used paraffin wax (PW, crystallization temperature ∼53 °C) as a PCM because of its relatively high phase change enthalpy, appropriate phase transition temperature, low cost, and high stability. We first prepared Fe_3_O_4_ incorporated-EG (EG@Fe_3_O_4_) and then combined it with PW and FS to form PW/FS/EG@Fe_3_O_4_ CPCMs with varying PW contents (60, 70, 75, and 80%). In the composites, the porous network of FS stabilized a large amount of PW without liquid leakage. Meanwhile, the electrothermal and magnetothermal conversion was achieved owing to the ultrahigh electric conductivity of EG and the superparamagnetic nature of Fe_3_O_4_, respectively. The electro/magneto-converted heat was stored in the CPCMs during the melting of PW and then applied for practical thermotherapy during the crystallization, achieving sustained heat release for a desirable duration. This work provided new insights into the preparation and multifunctional energy conversion and storage of PW/FS/EG@Fe_3_O_4_ CPCMs toward wearable thermotherapy applications.

## 2. Materials and characterization methods

### 2.1 Materials

Fumed silica (Aerosil 200) was bought from Evonik Operations (German). Expandable graphite was purchased from Sigma Aldrich (US). Paraffin wax (crystallization point ∼53 °C) was purchased from Sinopec Chemical (China). Iron(iii) chloride (FeCl_3_, AR), iron(ii) chloride tetrahydrate (FeCl_2_·4H_2_O, AR), ammoniac (NH_3_, 25–28%, AR), absolute ethanol, and hexane (AR) were purchased from Xylong Chemical (China).

### 2.2 Preparation of EG@Fe_3_O_4_

First, 0.1714 g FeCl_2_·4H_2_O and 0.1398 g FeCl_3_ were dissolved in a solution of water:ethanol (3.5 : 1 (v/v)) with the assistance of ultrasonication. Then, EG (0.1 g) was added to the solution, and the as-obtained mixture was stirred at ambient temperature for 1 hour using a glass rod. Next, the mixture was raised to 80 °C, and 20 mL of concentrated NH_3_ solution was slowly added until reaching pH ∼11 while continuing to stir using the glass rod. The black product was collected by filtration and washed with distilled water until reaching neutral pH. Finally, the mixture was dried in an oven at 120 °C for 12 h to gain EG@Fe_3_O_4_ with an EG : Fe_3_O_4_ ratio of 1 : 1 (w/w).

### 2.3 Preparation of PW/FS/EG@Fe_3_O_4_ CPCMs

The preparation of PW/FS/EG@Fe_3_O_4_ CPCMs was conducted using a well-known solvent-assisted method.^7^ First, a predetermined amount of PW was dissolved in hexane. Then, predetermined contents of EG@Fe_3_O_4_ and FS were added, and the mixture was stirred at ambient temperature for 2 hours using a glass rod. Afterward, the mixture was raised to 70 °C and stirred with the glass rod for the evaporation of hexane. The resulting sample was further treated in an oven at 70 °C for 12 hours, obtaining PW/FS/EG@Fe_3_O_4_ CPCMs with PW contents of 50–80 wt%. For comparison, a composite containing 75% PW and 25% FS was also prepared using the same procedure without the addition of EG@Fe_3_O_4_. The detailed compositions of the prepared composites and their abbreviated names are shown in Table 1.

**Table 1:** Detailed compositions of the prepared PW/FS/EG@Fe_3_O_4_ CPCMs

CPCM	Abbreviated name	PW (%)	FS (%)	EG@Fe_3_O_4_ (1 : 1 w/w) (%)
60% PW/FS/EG@Fe_3_O_4_	60% PW	60	26.7	13.3
70% PW/FS/EG@Fe_3_O_4_	70% PW	70	20.0	10.0
75% PW/FS/EG@Fe_3_O_4_	75% PW	75	16.7	8.3
80% PW/FS/EG@Fe_3_O_4_	80% PW	80	13.3	6.7
75% PW/FS	75% PW/FS	75	25	0

### 2.4 Characterization methods

The morphology was observed using scanning electron microscopy (SEM, Hitachi S-4800, Japan). The porous properties were characterized using N_2_ adsorption–desorption isotherm (MicroActive TriStar II Plus 2.03). The functional groups were scanned using Fourier-transform infrared spectroscopy (FTIR, JASCO FTIR 4600, Japan) within a wavenumber range of 4000–400 cm^−1^. The crystallization properties were determined using X-ray diffraction (XRD, Empyrean Diffractometer, Panalytical) within a 2*θ* range of 5–80°. The phase change properties were determined using differential scanning calorimetry (DSC, 214 Polyma, NETZSCH, German), which was performed under N_2_ gas of 20 mL min^−1^, a heating rate of 5 °C min^−1^, and a temperature range from 0 to 80 °C. The thermal stability was analyzed using thermogravimetric analysis (TGA, Labsys Evo TG-DSC 1600 analyzer, Setaram Instrument) within a temperature range of 30–800 °C with a heating rate of 10 °C min^−1^ under N_2_ gas of 20 mL min^−1^. Thermal conductivity was determined by a TPS 3500 instrument (Hot Disk AB, Sweden).

## 3. Results and discussion

### 3.1 Characterization

**Fig. 1:**
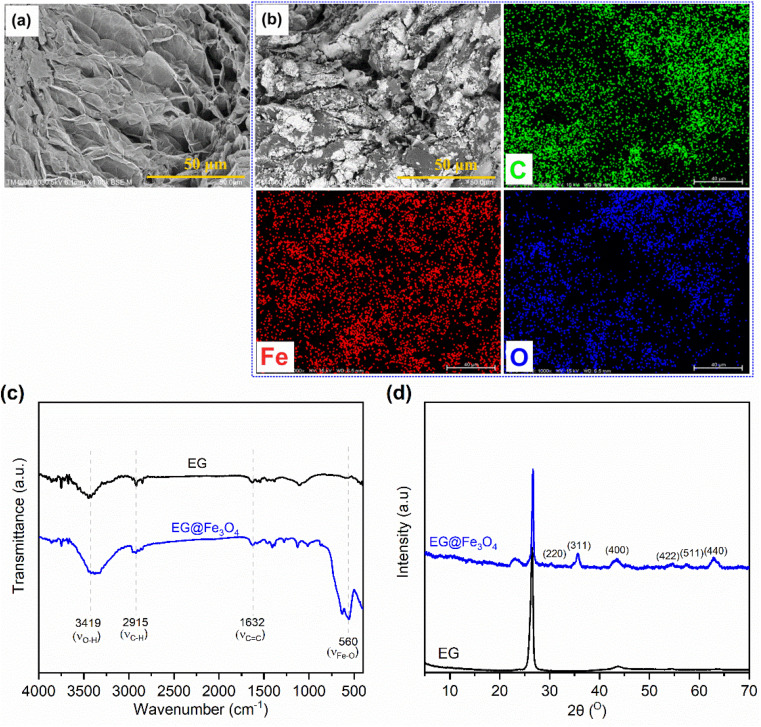
(a) SEM image of pristine EG, (b) SEM image and corresponding EDS elemental mapping images of EG@Fe_3_O_4_, (c) FTIR spectra of pristine EG and EG@Fe_3_O_4_, and (d) XRD patterns of pristine EG and EG@Fe_3_O_4_.

Fig. 1(a and b) exhibits SEM images of pristine EG and the prepared EG@Fe_3_O_4_. EG (Fig. 1a) consisted of stacked graphite sheets, forming a three-dimensional (3D) porous structure. After the modification with Fe_3_O_4_ (Fig. 1b), the surfaces of EG's sheets were densely attached by Fe_3_O_4_ particles, which was further characterized by the presence of Fe and O beside C elements in its corresponding EDS elemental mapping images (Fig. 1b). The FTIR spectrum of EG@Fe_3_O_4_ (Fig. 1c) fully exhibited characteristic absorption peaks of pristine EG, *i.e.*, peaks at 3419, 2915, and 1632 cm^−1^ assigned to vibrations of surface O–H groups, C–H groups, and C=C groups, respectively.^20,21^ In addition, a new absorption peak at 560 cm^−1^ appeared, assigned to the vibrations of Fe–O groups.^22^ The XRD pattern of EG@Fe_3_O_4_ (Fig. 1d) also exhibited a characteristic diffraction peak of pristine EG at 26.5° and new peaks at 30.3, 35.8, 43.7, 54.7, 57.6, and 63.1° corresponding to planes (220), (311), (400), (422), (511), and (440) of magnetic Fe_3_O_4_ nanoparticles.^17,23^ Raman spectroscopy results also presented the presence of Fe_3_O_4_ in EG (see Section 1 in the ESI†). The SEM, FTIR, and XRD results successfully demonstrated a combination of EG and Fe_3_O_4_.

**Fig. 2:**
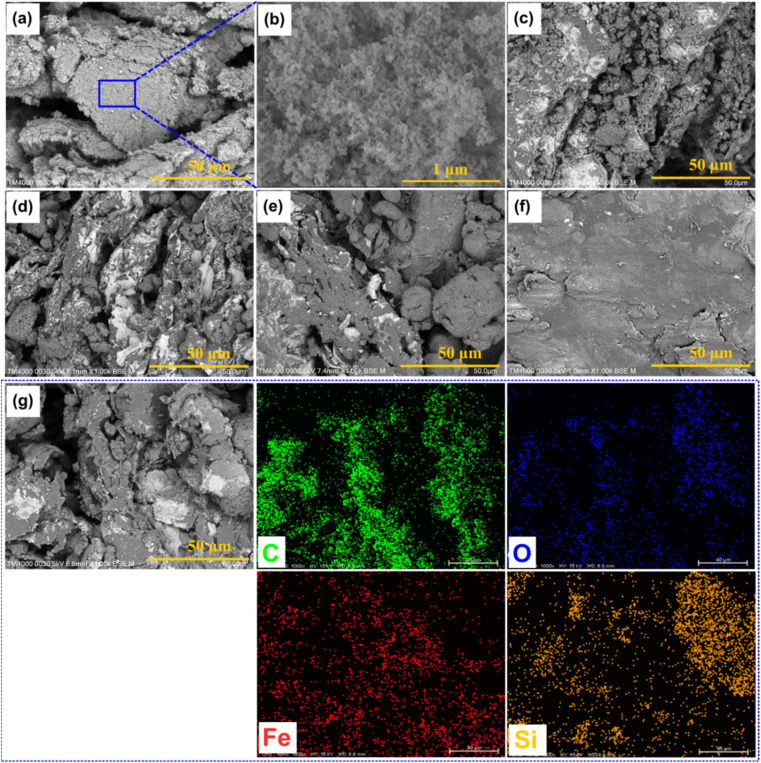
SEM images of (a and b) FS at different magnifications, (c–f) 60, 70, 75, and 80% PW CPCMs, and (g) EDS elemental mapping images of the 75% PW CPCM.

**Fig. 3:**
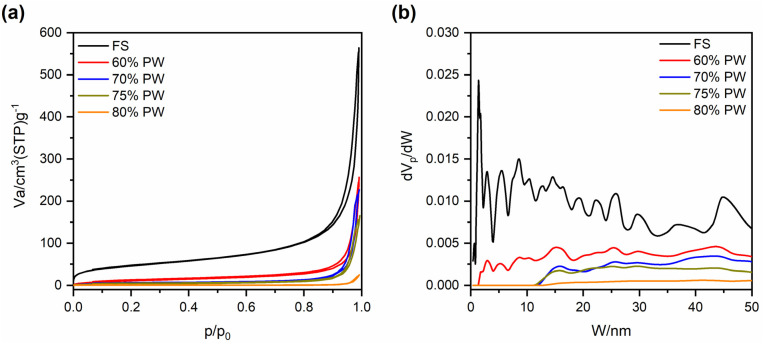
(a) N_2_ adsorption–desorption isotherms and (b) relevant pore size distribution of pristine FS and 60, 70, 75, and 80% PW CPCMs. Note that the data of pristine FS was taken from our previous report.^24^

Fig. 2(a and b) exhibits the SEM images of pristine FS at different magnifications, from which it can be seen that FS was composed of SiO_2_ nanoparticles aggregated into a 3D porous structure. The N_2_ adsorption–desorption isotherm of FS and its relevant pore size distribution (PSD) are shown in Fig. 3, while the specific textural properties are shown in Table S1.† The surface area of FS was 205 m^2^ g^−1^, its pore sizes were in the range of micro and mesopores, and its micro–mesopore volume was 0.95 cm^3^ g^−1^. In addition, FS also consisted of macropores within 50–150 nm and a total pore volume of 17 m^3^ g^−1^, as obtained from the mercury intrusion porosimetry in our previous report.^25^ The large pore volume and interconnected system of micro–meso–macro pores of FS provided sufficient space and transport pathways for the infiltration of PCMs. FS was combined with PW and EG@Fe_3_O_4_ to form composite phase change materials with electro and magnetothermal conversion and storage ability. Fig. 2c–f shows the SEM images of PW/FS/EG@Fe_3_O_4_ CPCMs with 60–80% PW. FS was mixed with EG@Fe_3_O_4_ in the CPCMs, and their surfaces were gradually covered with PW with increasing PW contents. The EDS elemental mapping images of 75% PW sample confirmed the presence of all C, O, Fe, and Si elements, as shown in Fig. 2g. The N_2_ sorption isotherms (Fig. 3a) and corresponding PSDs (Fig. 3b) of 60–80% PW CPCMs exhibited a gradual decrease in N_2_ adsorption and disappearance of pores, indicating PW was infiltrated in the pores of FS. Raman spectroscopy results also demonstrated the confinement of PW in FS and EG@Fe_3_O_4_ pores (see Section 1 in the ESI†). Notably, FS and EG@Fe_3_O_4_ could not be seen clearly in the 80% PW CPCM due to the complete covering of PW (Fig. 2f), suggesting there was a surplus amount of PW at this PW content.

**Fig. 4:**
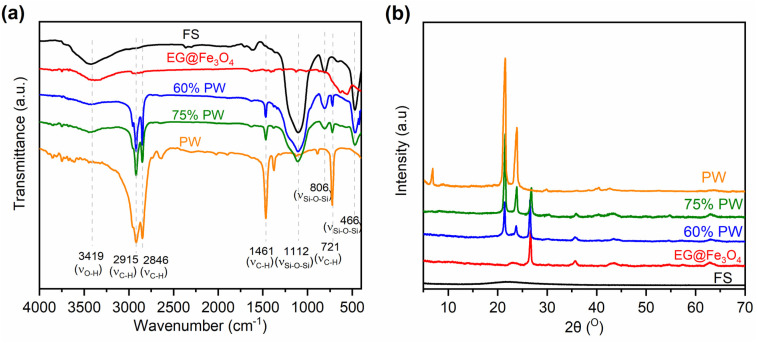
(a) FTIR spectra of pristine FS, EG@Fe_3_O_4_, pristine PW, 60% PW, and 70% PW CPCMs, and (b) XRD patterns of the corresponding materials.

Fig. 4a exhibits the FTIR spectra of two representative CPCMs (60% and 75% PW) compared to those of pristine PW, FS, and EG@Fe_3_O_4_. The two CPCMs fully combined the characteristic absorption peaks of the pristine components. For example, those originating from FS were found at 3419 cm^−1^ assigned to the stretching vibrations of surface –O–H groups, and 1112, 806, and 466 cm^−1^ assigned to the asymmetric stretching, symmetric stretching, and bending vibrations of the Si–O–Si groups, respectively FS.^13^ Meanwhile, the absorption peaks inherited from pristine PW could be seen at 2915, 2846, 1461, and 721 cm^−1^, assigned to the vibration modes of C–H groups.^26^ Notably, no new peaks appeared in the spectra of the two CPCMs. Similarly, the XRD patterns of the two CPCMs (Fig. 4b) fully exhibited the characteristic diffraction peaks of pristine PW at 21.5 and 23.9° and of EG at 26.5°. It is noted that the diffraction peaks of Fe_3_O_4_ could not be seen clearly in the two CPCMs because of their inherently low intensities. The FTIR and XRD results indicated that FS, PW, and EG@Fe_3_O_4_ were physically integrated, and their crystallization properties were intact in the CPCMs.

### 3.2 Phase change behaviors

**Fig. 5:**
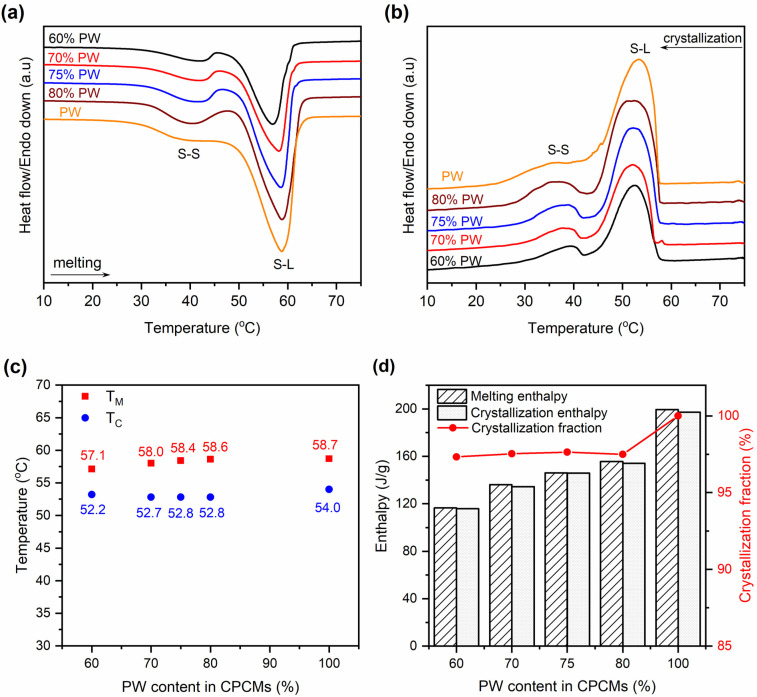
(a and b) Melting and crystallization curves of pristine FS and the prepared 60–80% PW CPCMs, (c) melting and crystallization temperatures of the corresponding materials, and (d) melting and crystallization enthalpies and crystallization fractions of the corresponding materials.

The phase change behaviors of the prepared 60–80% PW CPCMs compared to pristine PW were characterized by DSC, and the obtained thermograms are shown in Fig. 5(a and b). Both pristine PW and the CPCMs showed two peaks during the melting and crystallization processes. The small peaks at lower temperatures were attributed to solid–solid (S–S) phase transitions, while the larger ones at higher temperatures were due to the solid–liquid (S–L) phase transitions of PW,^27,28^ and the detailed S–L transition temperatures are shown in Fig. 5c. Compared to pristine PW, the 60–80% PW CPCMs exhibited slightly lowered S–L phase transition temperatures of 0.1–1.6 °C during the melting processes and 1.2–1.8 °C during the crystallization processes. This was consistent with previous reports showing that the phase change temperatures of PCMs in the form of CPCMs were reduced due to confinement effects and physical interactions with porous supports.^29,30^ The lowered phase change temperatures were also observed when other paraffin-based^31^ and non-paraffin-based PCMs such as stearic acid,^24^ CH_3_COONa·3H_2_O,^13^ and 1-octadecanol^32^ were confined in FS.

The melting/crystallization phase change enthalpies (Δ*H*_M_/Δ*H*_C_) of the 60–80% PW CPCMs and pristine PW were presented in Fig. 5d. The CPCMs exhibited lower Δ*H*_M_/Δ*H*_C_ than pristine PW and their Δ*H*_M_ and Δ*H*_C_ increased with the increasing PW contents, ranging from 116.5 and 115.8 J g^−1^ for 60% PW CPCM to 155.6 and 154.2 J g^−1^ for 80% PW one, respectively. These results could be readily understood as the phase change enthalpies of the CPCMs solely came from the melting and crystallization of PW, while the other components, including FS and EG@Fe_3_O_4_, made no contribution. Thus, an increase in PW content in the CPCMs induced greater phase change enthalpies. Another important parameter affecting the phase change enthalpies is the crystallization fraction (*F* (%)) of the confined PW in CPCMs, which could be calculated using eqn (1):^29^1
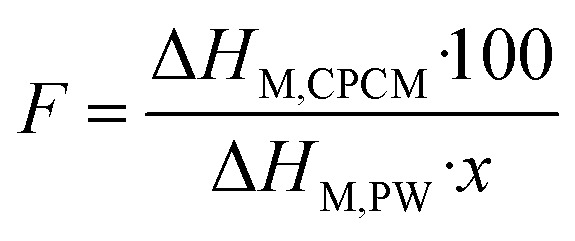
where Δ*H*_M,CPCM_ and Δ*H*_M,PW_ are the melting enthalpies of the CPCMs and pristine PW, respectively, and *x* is the weight fraction of PW in the CPCMs. As shown in Fig. 5d, the 60–80% PW exhibited consistently high crystallization fractions of 97.3–97.5%. The crystallization fraction of a PCM confined in porous supports greatly depends on interfacial interactions between the PCM and surface functional groups of the porous supports. Literature showed that interfacial hydrogen bonding interactions between silanol groups on SiO_2_'s surfaces and PCMs such as alcohols, fatty acids, polyols, and salt hydrates restricted the free mobility of the PCMs, thus declining the crystallization fractions.^13,32,33^ For example, the crystallization fractions were found to be only 91.2% for 1-octadecanol, 86% for CH_3_COONa·3H_2_O, or even 0% for PEG, as they were confined in SiO_2_-based porous supports (Table 2). The declined crystallization fractions significantly lowered the heat storage capacities of the composites. In this work, the crystallization fraction of PW confined in FS was superior to most of the reported values because PW was unable to form interfacial hydrogen bonds with FS. Thus, the advantage of using PW as a PCM over fatty alcohols, fatty acids, polyols, and salt hydrates is the sustaining of high crystallization fractions, benefiting the heat storage capacity. The crystallization fraction of PW confined in FS and EG@Fe_3_O_4_ in this work also surpassed those of PW confined in other porous supports, including aerogel, nanoscroll, EG@SiO_2_, melamine foam, and sepiolite, with their *F* values in the range of 77.4–96.1% (Table 2). It is noted that the crystallization fractions (97.3–97.5%) of PW in the CPCMs could not reach 100% because the combination with solid substances (FS and EG@Fe_3_O_4_) still somehow restricted PW from a perfect crystallization as in the bulk.

**Table 2:** Thermal properties of the prepared 75% PW/FS/EG@Fe_3_O_4_ CPCM compared to other reported CPCMs

CPCM	Δ*H*_M_ (J g^−1^)	*F* (%)	TC/TC enhancement^a^ (W per (m K)) per times	Ref.
70% 1-octadecanol/FS	147.2	91.2	—	32
70% CH_3_COONa·3H_2_O/FS	132.6	86	0.560/0.87	13
70% PEG/SiO_2_	0	0	—	33
99.4% PW/aerogel	141.8	77.4	0.3261/1.4	26
60% PW/nanoscroll	128.5	96.1	0.52/2.0	28
74% PW/EG/SiO_2_	103.4	84.8	2.053/8.5	34
97.5% PW/melamine foam	130.6	87.8	0.56/2.0	35
35.2% PW/sepiolite	60.1	94.3	—	36
75% PW/FS/EG@Fe_3_O_4_^b^	146.1	97.6	1.648/7.3	This work

a Thermal conductivity/thermal conductivity enhancement compared to pure PCM.

b The 75% PW/FS/EG@Fe_3_O_4_ was selected for comparison because it was considered the optimal CPCM (see Section 3.4).

### 3.3 Thermal stability, thermal conductivity, leakage resistance, and cycling durability

**Fig. 6:**
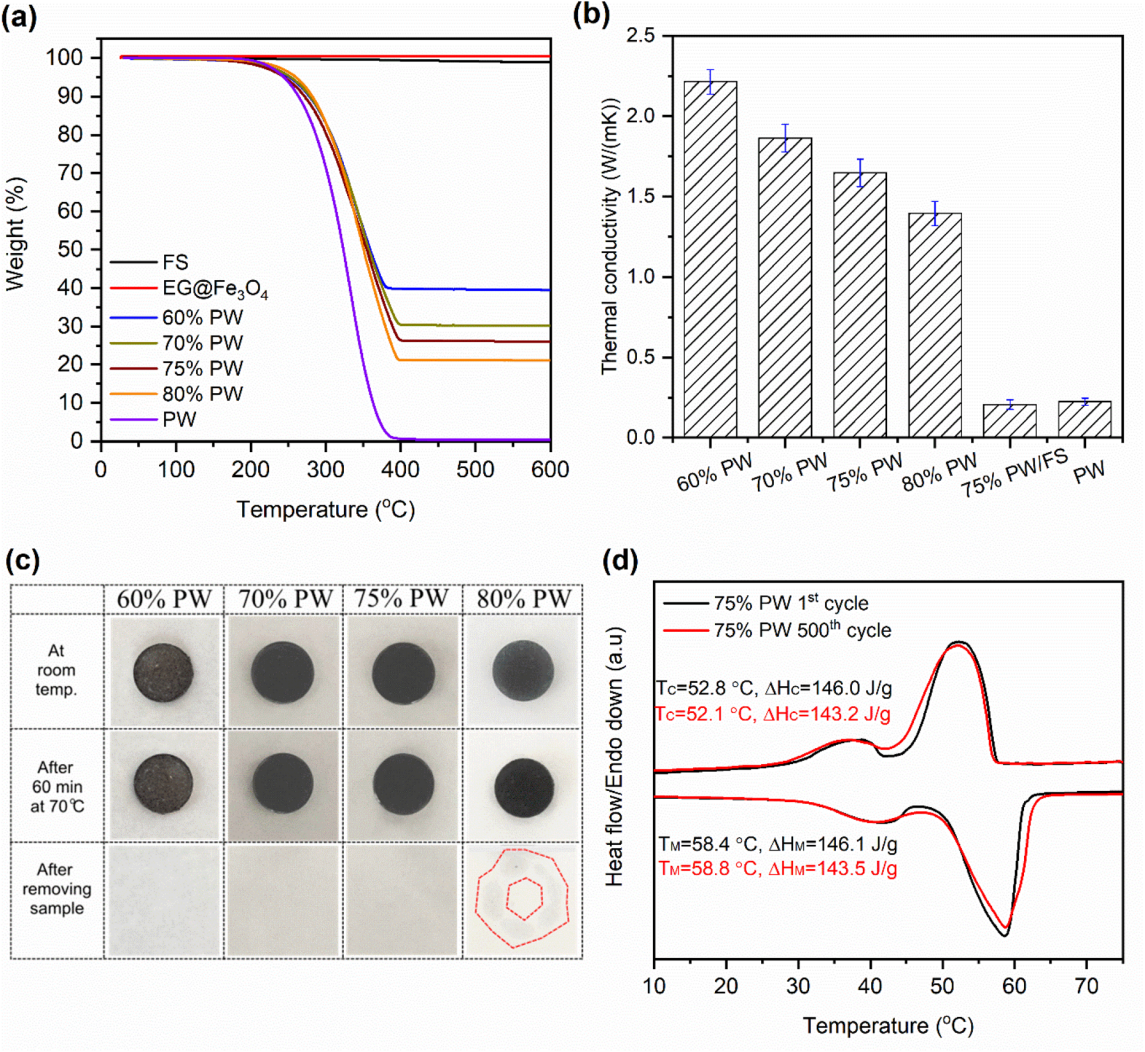
(a) TGA curves of pristine FS, EG@Fe_3_O_4_, 60–80% PW CPCMs, and pristine PW, and (b) thermal conductivities of 60–80% PW CPCMs and pristine PW, (c) digital photos of 60–80% PW CPCMs during the leakage test, and (d) DSC curves of 75% PW CPCM before and after 500 melting/crystallization cycles.

The thermal stability of the prepared 60–80% PW CPCMs compared to pristine FS, PW, and EG@Fe_3_O_4_ was analyzed by TGA, and the results are exhibited in Fig. 6a. FS and EG@Fe_3_O_4_ showed insignificant weight loss at up to 600 °C, indicative of excellent thermal stability. Meanwhile, PW was decomposed within 271–370 °C with almost 100% weight loss. The CPCMs exhibited better thermal stability than pure PW, characterized by a thermal composition temperature range of approximately 281–399 °C. This phenomenon was due to forces such as capillary and surface tension that hampered the spillover of PW from the pores of FS and EG@Fe_3_O_4_ before decomposition, thus promoting the stability of the CPCMs. This result demonstrated that once residing in the FS and EG@Fe_3_O_4_ porous networks, the surrounding inorganic supporters improved the thermal stability of PW as it was subjected to extreme conditions (*i.e.*, elevated temperatures).

The thermal conductivities of the 60–80% PW/FS/EG@Fe_3_O_4_ CPCMs compared to pristine PW are shown in Fig. 6b. Pristine PW exhibited a low thermal conductivity of 0.225 W (m K)^−1^. Notably, the 60–80% PW CPCMs possessed enhanced thermal conductivities of 2.215–1.395 W (m K)^−1^, which were 9.8–6.2 times greater than that of pristine PW. Of the components of the prepared PW/FS/EG@Fe_3_O_4_ CPCMs, FS was known to have extremely low thermal conductivity (∼0.045 W (m K)^−1^) because of the absence of a convection factor, caused by the similarity in its pore sizes and the mean free path (70 nm) of air.^37,38^ Previous reports showed that combining with FS even caused a decrease in the thermal conductivity of PCMs.^13,39^ Here, for confirmation, a composite containing 75% PW, 25% FS, and 0% EG@Fe_3_O_4_ (abbreviated as 75% PW/FS) was prepared and characterized for thermal conductivity. Compared to the thermal conductivity of pristine PW (0.225 W (m K)^−1^), that of the 75% PW/FS was decreased to 0.207 W (m K)^−1^ (Fig. 6b), consistently confirming the negative effect of FS on the PCM's thermal conductivity. Fe_3_O_4_ particles were also known to have a low thermal conductivity of 0.144–0.18 W (m K)^−1^.^40,41^ Therefore, the enhanced thermal conductivity of the prepared PW/FS/EG@Fe_3_O_4_ CPCMs was attributed to EG's extremely high thermal conductivity (25–470 W (m K)^−1^).^42–45^ This phenomenon was also observed elsewhere, showing thermal conductivity enhancements of 7.16–17.3 times for CPCMs with the addition of 6–15 wt% EG.^42–44^ As shown in Table 2, the thermal conductivities of PW/FS/EG@Fe_3_O_4_ CPCMs were comparable to or even surpassed those of other PW-based CPCMs including PW/aerogel (0.3261 W (m K)^−1^), PW/nanoscroll (0.52 W (m K)^−1^), PW/EG@SiO_2_ (2.053 W (m K)^−1^), and PW/melamine foam (0.56 W (m K)^−1^), with the corresponding thermal conductivities shown in the paratheses.

The leakage resistance of the 60–80% PW/FS/EG@Fe_3_O_4_ CPCMs was examined by treating them at 70 °C (∼12 °C above their melting points) for 60 min. The CPCMs were subsequently carefully observed for leakage stains, and the digital photos taken during the test are shown in Fig. 6c. The 60–75% PW CPCMs showed unnoticeable leakage after the thermal treatment, indicating good leakage resistance of up to 75% PW. This was due to the confinement of PW in the interconnected pores of FS and EG@Fe_3_O_4_ by the capillary and surface tension forces, which were capable of preventing PW from leakage even in its liquid state. Previous reports demonstrated that FS could stabilize up to 70–80% of various PCMs.^13–15^ In addition, the EG, with its 3D porous network, was also a good adsorbent for PCMs, thus contributing to the adsorption and stabilization of PW.^34,46^ Notably, the 80% PW CPCM was subjected to liquid leakage, which indicated an excessive PW content.

The prepared PW/FS/EG@Fe_3_O_4_ CPCMs were able to retain up to 75% PW without leakage, the 75% PW sample was thus selected as the optimal composite for further investigations. The cycling durability or so-called thermal reliability of the 75% PW was examined for 500 phase change cycles, and the DSC thermograms at the first and 500th cycles are shown in Fig. 6d. The DSC curves showed negligible change after the multiple thermal cycles. The *T*_M_ and *T*_C_ were altered by only 0.4 and 0.7 °C, respectively. In addition, the Δ*H*_M_ and Δ*H*_C_ after 500 thermal cycles reached 143.5 and 143.2 J g^−1^, which were only 1.8 and 1.9% lower than those of the first cycles, respectively. These results demonstrated that the 75% PW CPCM possessed excellent cycling durability, making it suitable for long-term utilization.

### 3.4 Magnetothermal conversion and storage

**Fig. 7:**
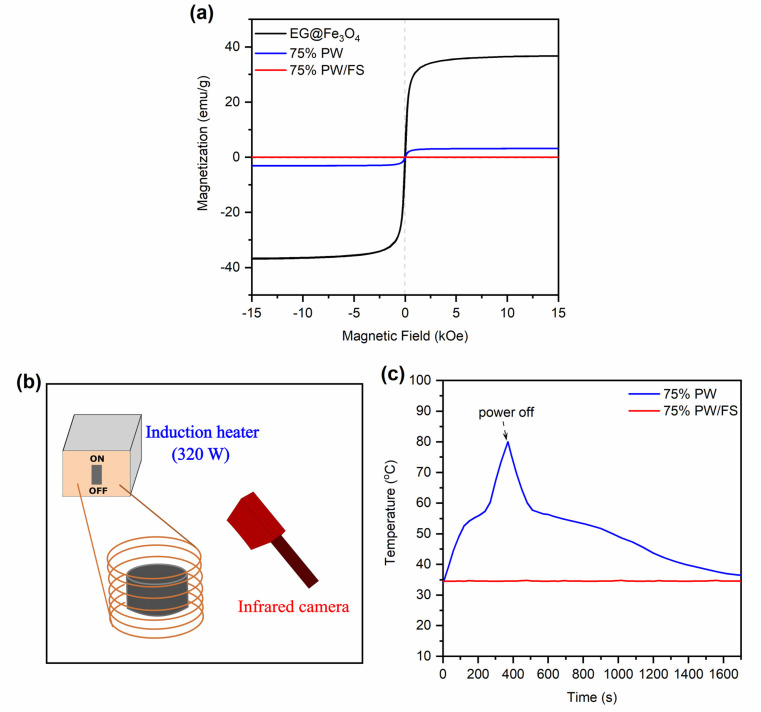
(a) VSM curves of EG@Fe_3_O_4_, 75% PW/FS/EG@Fe_3_O_4_ CPCM, and 75% PW/FS sample, (b) illustration of magnetothermal conversion apparatus, and (c) temperature–time curves of 75% PW/FS/EG@Fe_3_O_4_ CPCM and 75% PW/FS sample during magnetothermal conversion and storage experiment.

Fig. 7a shows the VSM curves of the prepared 75% PW/FS/EG@Fe_3_O_4_ CPCM compared to those of EG@Fe_3_O_4_ and 75% PW/FS samples. The EG@Fe_3_O_4_ and 75% PW/FS/EG@Fe_3_O_4_ CPCM (containing 4.1% Fe_3_O_4_) possessed saturation magnetizations of 36.8 and 3.1 emu g^−1^. In addition, they showed very small coercivity and remanence, indicative of superparamagnetism. Meanwhile, the 75% PW/FS sample exhibited zero saturation magnetization, readily attributed to its non-magnetic nature.

Magnetic compounds can be heated in an alternating magnetic field due to the effects of Néel and Brownian relaxation effects.^16,47^ The combination of CPCMs with magnetic materials allows the CPCMs to convert magnetic energy into heat and store it in the CPCMs due to the high phase change enthalpies, thus providing an effective charging method for the CPCMs. Here, an alternating magnetic field of 320 W was applied to the 75% PW/FS/EG@Fe_3_O_4_ CPCM to evaluate the magnetothermal conversion and storage performance (Fig. 7b), and the temperature evolutions during the test are shown in Fig. 7c. The applied magnetic field was effectively converted into thermal energy, making the temperature of 75% PW/FS/EG@Fe_3_O_4_ CPCM rapidly increase from 33.8 to 80.1 °C in 167 s. The temperature growth during the test are divided into three phases. In the first phase (33.8–52.5 °C), the CPCM exhibited a fast temperature evolution because the magnetic energy was stored as sensible heat. The second phase (52.5–58.3 °C) was accompanied by the melting of PW, storing the magnetic energy as latent heat, and forming a temperature platform. After the melting was completed, the CPCM went to the last phase (58.3–80.1 °C) where the magnetic energy was again stored as sensible heat, generating rapid temperature evolution. When the magnetic field was switched off, the CPCM exhibited a rapid temperature decrease due to a high-temperature gap with ambient temperture (∼33 °C). A temperature platform could be observed at approximately 55–48 °C during the cooling, indicative of the crystallization (heat release) of the CPCM. For comparison, the 75% PW/FS sample showed no temperature change when applied with the same magnetic power due to its non-magnetism. These results demonstrated good magnetothermal conversion and storage for the prepared 75% PW/FS/EG@Fe_3_O_4_ CPCM.

### 3.5 Electrothermal conversion and storage

**Fig. 8:**
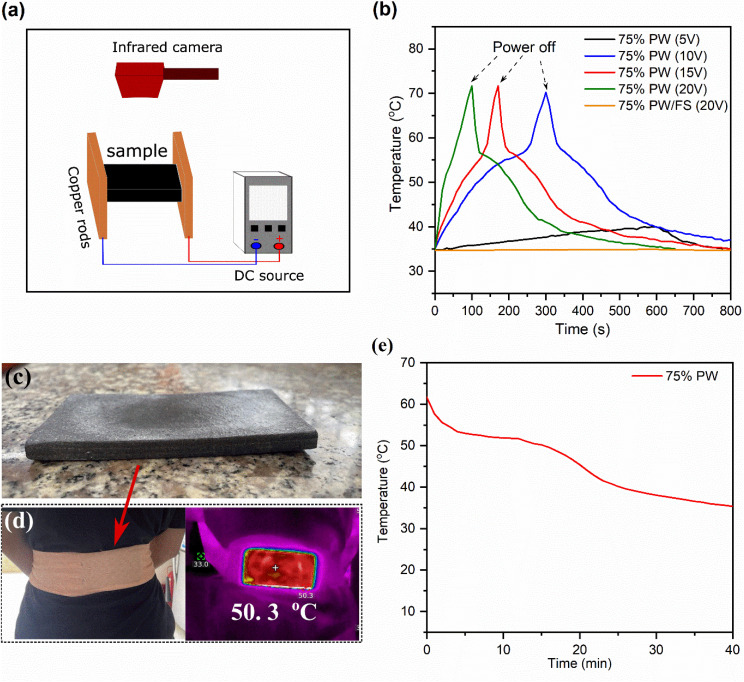
(a) Illustration of electrothermal conversion apparatus, (b) temperature–time curves of 75% PW/FS/EG@Fe_3_O_4_ CPCM at different applied voltages and 75% PW/FS sample at an applied voltage of 20 V, (c) digital photo of the prepared 75% PW/FS/EG@Fe_3_O_4_ heat pack, (d) digital and infrared photos of the 75% heat pack when applied to the back of a volunteer, and (e) temperature–time curve at the center of the pack's inner face during the test.

Due to the high availability and low cost of electricity networks worldwide, CPCMs possessing an electrothermal conversion ability can be easily heated by electricity, which is highly satisfactory for thermotherapy applications that require a fast and effective charge. Here, the electrothermal conversion and storage of the 75% PWPW/FS/EG@Fe_3_O_4_ CPCM were examined by applying a DC source at different voltages (5–20 V). The temperature evolutions during the experiment were obtained using an infrared camera, as illustrated in Fig. 8a, and the obtained temperature–time curves are exhibited in Fig. 8b. With an applied voltage of 5 V even for 600 s, the temperature of the CPCM only increased from ∼35 to 39.8 °C, which was below the melting temperture of the CPCM. This suggested that 5 V was not sufficient to charge the CPCM. With increasing the applied voltages to 10, 15, and 20 V, the temperatures of the CPCM could surpass its melting point, reaching 70 °C after 300, 161, and 90 s, respectively. The electrothermal conversion ability of the 75% PWPW/FS/EG@Fe_3_O_4_ CPCM was attributed to the EG's extremely high electrical conductivity (∼10 000 S cm^−1^)^48^ and complied with Joule's law, which states that an electrical flow passing a material generates heat.^1,49^ For comparison, the 75% PW/FS showed no temperature evolution even at an applied voltage of 20 V (Fig. 8b) due to the poor conductivity of both PW and FS (∼10^−16^–10^−12^ S cm^−1^).^50,51^

The temperature evolutions of the 75% PW/FS/EG@Fe_3_O_4_ CPCM at applied voltages of 10, 15, and 20 V are divided into three phases. The first phase (35.0–51.0 °C) was accompanied by the conversion of electric energy into thermal energy, which was stored in the CPCM as sensible heat, leading to a rapid temperature increase. In the second phase (51–56 °C), the converted thermal energy was stored as latent heat during the melting of PW in the CPCM, characterized by a temperature plateau. In the final phase (56–70 °C) after the melting was completed, the CPCM exhibited a rapid temperature increase because the applied energy was stored as sensible heat. After the power was switched off and the material was left to cool naturally at an ambient environment (∼34 °C), its temperature went down quickly and then established temperature plateaus at ∼55–45 °C because of the crystallization (heat release) of PW. These results demonstrated good electrothermal conversion and thermal storage of the 75% PW/FS/EG@Fe_3_O_4_ CPCM.

### 3.6 Practical thermotherapy performance

CPCMs can be applied to thermotherapy applications during their crystallization processes, which release latent heat at nearly constant temperatures for certain durations. In this work, the 75% PW/FS/EG@Fe_3_O_4_ CPCM possessed a crystallization temperature of 52.8 °C, thus being applicable for high-temperature thermotherapies that require temperatures within 50–55 °C. To study the practical efficacy, the 75% PW CPCM (30 g) was compacted into a heat pack (110 × 55 × 5 mm) (Fig. 8c) and then electrically charged at 20 V to ∼65 °C to store the melting latent heat. The thermotherapy heat pack can be practically used to treat any body surface requiring a thermal treatment, *e.g.*, the shoulder, the back, or the neck. Here, the heat pack was applied to the back of a volunteer (Fig. 8d), and the temperature variations at the center of the pack's inner face contacting with the volunteer's back were monitored using a thermocouple. As shown in Fig. 8e, the heat pack sustained the temperature within 50–55 °C for 12 min, which was due to the release of heat during the crystallization of PW. Compared to the high-temperature thermotherapy criteria that require sustaining temperatures within 50–55 °C for 6 min,^3^ the 75% PW heat pack meets or even surpasses the requirement, thus being suitable for actual applications.

### 3.7 Future perspective

The overall performance of a CPCM in practical thermal energy storage applications is influenced by various parameters, including phase change temperature, phase change enthalpy, thermal conductivity, material mass, geometry, density change during solid–liquid transition, and ambient conditions (temperature and wind flow), *etc.* Previous reports demonstrated that the prediction and optimization of these parameters can be conducted using numerical analyses, saving human forces for experimental investigations. Zhang *et al.*^52^ conducted a numerical study on the performance of a CPCM for a thermotherapy mask. Liu *et al.*^53^ investigated the thermal performance of lightweight building walls incorporated with a CPCM using a numerical analysis. Wu *et al.*^54^ used a numerical analysis to study the solar energy storage of a CPCM. Recently, artificial intelligence (AI) tools have also been applied for the prediction and optimization of CPCMs in various applications, including molten salt incorporated-solar thermal power plants^55,56^ and CPCM-based concrete for building energy saving.^57,58^ Results showed that AI tools could provide quite exact predictions of the performance of CPCMs. In this work, although the prepared PW/FS/EG@Fe_3_O_4_ CPCMs were experimentally proven to be applicable in thermotherapy, the practical utilization still needs more investigations and optimizations. Therefore, we suggest using numerical simulations or AI tools to solve this issue in the future.

## 4. Conclusion

The PW/FS/EG@Fe_3_O_4_ CPCMs were successfully prepared and studied for thermal properties and multifunctional thermal conversion and storage with varying PW contents of 60–80%. The 60–80% PW CPCMs exhibited high phase change enthalpies, ranging from 116.5 J g^−1^ for the 60% PW sample to 155.6 J g^−1^ for the 80% PW one, which was facilitated by high crystallization fractions (above 97%) of confined PW. The thermal conductivities of the prepared CPCMs were achieved at 1.395–2.215 W (m K)^−1^, which were 9.8–6.2 times greater than that of pristine PW. In addition, the leakage resistance test demonstrated a large amount of PW (75%) could be stabilized in the CPCMs. A cycling durability test also demonstrated that the 75% PW CPCM maintained its phase change behaviors even after 500 melting/crystallization cycles. In addition, the 75% PW CPCM exhibited a saturation magnetization of 3.1 emu g^−1^ and inherited the superparamagnetism from Fe_3_O_4_. This offered the CPCM a magnetothermal conversion and storage, showing a temperature increase from 33.8 to 80.1 °C in 370 s as applied with an alternating magnetic field of 320 W. The high electrical conductivity of EG further provided the CPCM with an electrothermal conversion and storage, showing a temperature increase from 34.7–71.7 °C in 100 s as applied with a DC voltage of 20 V. Moreover, the 75% PW, in the form of a heat pack, demonstrated a sustained heat release when applied to practical thermotherapy. The good thermal properties and electro/magnetothermal conversion and storage ability make the prepared CPCMs promising for practical thermotherapy applications.

## Ethical statement

The ethics responsibility is informed and acknowledged to all the authors and volunteers as a part of content of right of informed consent.

## Data availability

The data supporting this article have been included as part of the ESI.†

## Conflicts of interest

The authors declare no known competing interests.

## Supplementary Material

RA-015-D5RA00438A-s001
