# Supplementary material for: Fumed silica-based composite phase change materials with effective electric and magnetic heating abilities for wearable thermotherapy
Source: RSC Adv. 2025 Apr 11;15(15):11465–77. doi: 10.1039/d5ra00438a (PMC11987084; doi:10.1039/d5ra00438a)
Supplement: RA-015-D5RA00438A-s001 [file RA-015-D5RA00438A-s001.pdf]

This journal is © The Royal Society of Chemistry 2025

Supplemental information

Fumed Silica-Based Composite Phase Change Materials with Effective Electric and Magnetic Heating Abilities for Wearable Thermotherapy

1. Raman results

The Raman spectrum of EG@Fe_3O_4 compared to pristine EG is shown in Figure S1a. The pristine EG showed two features at 1582 and 2723 cm^{-1}, assigned to the G and 2D bands, which are typical for graphitic materials. The spectrum of EG@Fe_3O_4 additionally showed a feature of Fe_3O_4 at 716 cm^{-1} [1, 2], demonstrating the Fe_3O_4 inclusion. Figure S1b compares the Raman spectra of 60 and 75% PW CPCMs to FS, EG@Fe_3O_4, and pure PW. The Raman bands of pure PW have been well-documented in previous reports [3]. The features at 2850 and 2881 cm^{-1} were assigned to symmetric and asymmetric CH_2 stretching, respectively. Meanwhile, two weak shoulders at 2935 and 2963 cm^{-1} were assigned to symmetric and asymmetric CH_3 stretching, respectively. In addition, the feature at 1293 cm^{-1} was assigned to the CH_2 twisting band and those between 1400-1500 cm^{-1} were assigned to the CH_2 bending and scissor. In the C–C stretching region, the bands at 1061 and 1129 cm^{-1} were assigned to the symmetric and asymmetric C–C stretching, respectively. For the prepared 60 and 75% PW CPCMs, the vibrations of confined PW were altered compared to pure PW, in which the CH_2 vibration intensity at 2881 cm^{-1} was significantly decreased, and those between 1000 and 1500 cm^{-1} were not well resolved. This phenomenon was attributed to the confinement of PW inside FS and EG@Fe_3O_4 pores, limiting the stretching and vibrations of PW. These confinement effects were also discussed in previous reports [4-6]. Overall,

the Raman results demonstrated a successful preparation of EG@Fe_3O_4 and a successful infiltration of PW into FS and EG@Fe_3O_4 porous networks.

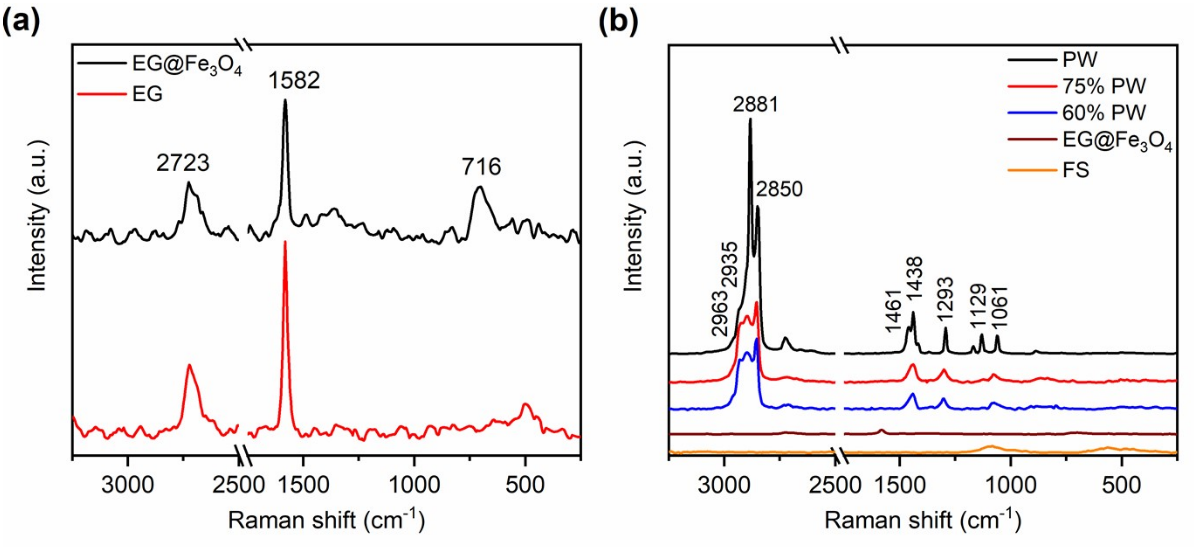

Figure S1. Raman spectra of (a) EG and EG@Fe_3O_4, and (b) PW, 75% PW, 60% PW, EG@Fe_3O_4, and FS.

2. Porosity properties

Table S1. Porous properties of FS and the prepared CPCMs

CPCMs	Surface area (m^2/g)	Pore volume* (cm^3/g)	Pore size (nm)
FS	205	0.95	-
60% PW	71	0.37	-
70% PW	26	0.32	-
75% PW	15	0.23	-
80% PW	-	0.035	-

*Pore volume calculated from the N_2 adsorption–desorption isotherm.

References

[1]. Guo C, Hu Y, Qian H, Ning J and Xu S 2011 Magnetite (Fe3O4) tetrakaidecahedral microcrystals: Synthesis, characterization, and micro-Raman study *Mater Charact* **62** (1)148-51.

[2]. de Jesús Ruíz-Baltazar Á, Reyes-López SY and Pérez R 2017 Magnetic structures synthesized by controlled oxidative etching: Structural characterization and magnetic behavior *Results in Physics* **7** 1828-32.

[3]. Zheng M and Du W 2006 Phase behavior, conformations, thermodynamic properties, and molecular motion of multicomponent paraffin waxes: A Raman spectroscopy study *Vib Spectrosc* **40** (2)219-24.

[4]. Wang J, Yang M, Lu Y, Jin Z, Tan L, Gao H, Fan S, Dong W and Wang G 2016 Surface functionalization engineering driven crystallization behavior of polyethylene glycol confined in mesoporous silica for shape-stabilized phase change materials *Nano Energy* **19** 78-87.

[5]. Aftab W, Huang X, Wu W, Liang Z, Mahmood A and Zou R 2018 Nanoconfined phase change materials for thermal energy applications *Energy Environ Sci* **11** (6)1392-424.

[6]. Gao H, Wang J, Chen X, Wang G, Huang X, Li A and Dong W 2018 Nanoconfinement effects on thermal properties of nanoporous shape-stabilized composite PCMs: A review *Nano Energy* **53** 769-97.
